# “You should have addressed it directly”: the ideals and ideologies of managing interaction problems in healthcare work

**DOI:** 10.1108/JHOM-01-2024-0006

**Published:** 2024-09-02

**Authors:** Elina Weiste, Melisa Stevanovic, Inka Koskela, Maria Paavolainen, Eveliina Korkiakangas, Tiina Koivisto, Vilja Levonius, Jaana Laitinen

**Affiliations:** Finnish Institute of Occupational Health, Helsinki, Finland; Faculty of Social Sciences, Tampere University, Tampere, Finland

**Keywords:** Discourse, Healthcare, Ideology, Open communication, Speaking up, Workplace interaction

## Abstract

**Purpose:**

An “open communication culture” in the workplace is considered a key contributor to high-quality interaction and providing means to address problems at work. We study how the ideals of “open communication” operate in healthcare.

**Design/methodology/approach:**

We use discourse analysis to investigate the audio-recorded data from 14 workshop team discussions in older people services.

**Findings:**

We found four imperatives concerning the interactional conduct of their colleagues in problematic situations that nursing professionals prefer: (1) Engage in direct communication and avoid making assumptions, (2) Address problems immediately, (3) Deal directly with the person involved in the matter and (4) Summon the courage to speak up. Through these imperatives, the nursing professionals invoke and draw upon the “open communication” discourse. Although these ideals were acknowledged as difficult to realize in practice and as leading to experiences of frustration, the need to comply with them was constructed as beyond doubt.

**Practical implications:**

Workplace communication should be enhanced at a communal level, allowing those with less power to express their perspectives on shaping shared ideals of workplace interaction.

**Originality/value:**

The expectation that an individual will simply “speak up” when they experience mistreatment by a colleague might be too much if the individual is already in a precarious position.

## Introduction

High-quality workplace interaction serves as the cornerstone of successful healthcare work, providing professionals with the means to address challenging situations and voice their ideas and concerns. An “open communication culture” in the workplace is a key contributor to a psychologically safe work environment and a climate of trust (e.g. Gur and Tzafrir, 2022; Anderson *et al.*, 2014), which can help multidisciplinary teams by establishing supportive and well-functioning teamwork. Well-functioning teams and a positive work environment reduce stress and enhance the work-related wellbeing of healthcare workers (Hämmig and Vetsch, 2021). Controversy, poor wellbeing, and burnout of healthcare workers are associated with reduced patient safety and quality of care (Hall *et al.*, 2016). Symptoms like fatigue, irritability, and reduced cognitive functioning may weaken individual work performance and put pressure on teamwork relations, causing a poorer safety climate (Hall *et al.*, 2016). This may further result in more distanced staff, poorer quality of care, and a higher risk of making errors. Open communication in teams may help professionals to detect these kinds of risks in time and prevent errors from reaching patients and causing them harm (Morrow *et al.*, 2016).

The “open communication culture” at work encompasses various ideals, including opportunities for continuous and open dialogue, fostering psychological safety and positive relationships among staff, and facilitating the exchange of knowledge (e.g. Anderson *et al.*, 2014). At heart of well-functioning team are interactions among its members (West and Markiewicz, 2016): colleagues are approachable, hold each other’s competence in high regard, express appreciation, actively seek and provide feedback, pay attention, ask questions, and help each other when needed (e.g. Bochatay, 2019; Espinoza *et al.*, 2018).

However, even within well-functioning teams, individuals frequently encounter problems in their interactions with colleagues. Managing such interactional problems in healthcare often involves navigating a complex landscape of diverse stakeholders, including team members, managers, patients, administrators, and policymakers. When problems occur, placing the patient at the forefront is fundamental. This entails looking at the situation from the patient’s perspective and understanding their needs, preferences, and values (e.g. Kurki *et al.*, 2024; Rathert *et al.*, 2016). Team members are expected to voice their concerns when they identify risky or unprofessional behaviors to prevent errors and ensure patient safety (Morrow *et al.*, 2016). Such risks include, for instance, rule breaking, failure to follow protocols, and erroneous clinical decisions. Also, rude or intimidating behaviors by team members are considered as a direct threat to the patient safety because they can prevent team members from speaking up (West and Markiewicz, 2016). “Speaking up” about problems is thus intrinsically connected to care quality (e.g. Bochatay, 2019; Espinoza *et al.*, 2018; Kane *et al.*, 2023; West and Markiewicz, 2016).

The field of healthcare, especially the nursing profession, is particularly intriguing in terms of the management of interactional problems at work. In this context, the ideal of “speaking up” is prevalent for two reasons. First, as nursing work primarily relies on communication, interaction skills are at the core of professional competence. Nursing professionals are expected not only to interact effectively with their patients but also to possess strong teamwork skills that are considered essential for ongoing professional development (Fox *et al.*, 2018). Consequently, nursing professionals are expected to be able to handle problematic interactional situations at work, of which the ability to “speak up” is a key aspect.

Second, being a “good nurse” often equates with “being a good person”. Nursing can be considered a moral practice, rooted in ethical obligations to promote and protect the fundamental good of another human being (Bliss *et al.*, 2017). The ideals of professionalism encompass adherence to a code of ethics and “an image of collegial work relations of mutual assistance and support … ” (Evetts, 2013, p. 788). Therefore, nursing goes beyond the mere execution of proficient interactions and skillful behavior. In addition, supporting colleagues and respecting their expertise become moral expectations, which also call for straightforward communication.

Despite the normative expectation to “speak up” about work -related problems, the opposite is more commonly the case in working life: remaining silent (e.g. Lam and Xu, 2019). The employees’ sense of being (un)able to voice their concerns is influenced by various factors. First, employees may feel uneasy about voicing their unfavorable observations due to fear of harming important relationships and jeopardizing trust and acceptance within their in-group (Morrow *et al.*, 2016; Morrison, 2023). “Speaking up” equals the delivery of bad news, which is something that people are hesitant to do, as it can disrupt the solidarity of meaningful relationships and their deliverer can be blamed as the source of the problem (Kitz *et al.*, 2023; Morrison, 2023). Consequently, for individuals, remaining silent about their concerns may seem to involve fewer psychosocial risks (Kritsotakis *et al.*, 2022). Second, the way in which people raise and handle problems is affected by the collective social norms and assumptions of teams and organizations. These norms either support or hinder “open communication,” as they guide people to infer how, when, and by whom problems can or should be voiced; what issues can be openly discussed within the community; and what issues should be kept silent (Morrow *et al.*, 2016; Lainidi *et al.*, 2023; Morrison, 2023). Teams may handle problems and difficult topics in significantly different ways: While some teams openly discuss problems to learn from them, others remain silent and avoid addressing them entirely (Kritsotakis *et al.*, 2022). In its most extreme form, organizations may develop “narratives of silence” where collective denial involves cumulative episodes of multilevel, dynamic failure that unfold and escalate over time, with multiple actors collectively engaging and complying to quash any dissenting voices (Hendy and Tucker, 2021). As Hendy and Tucker’s case analysis on the Mid Staffordshire hospital scandal in the UK indicates, such narratives of silence may lead to falling organizational standards on quality of care, unethical decision-making, and even brutal mistreatment of patients and an unacceptable number of patient deaths (Hendy and Tucker, 2021).

Teams’ capacities to discuss problems openly are also tied to their group characteristics and leadership styles. First, openness is facilitated by “psychological safety” (Frazier *et al.*, 2017) where employees believe that their colleagues will not reject them for speaking their minds (e.g. Sarfraz *et al.*, 2021). Second, employee voice and silence are affected by leadership styles and hierarchies at the workplace (Lam and Xu, 2019; Morrow *et al.*, 2016). Large power differentials and leadership focused on individual capabilities negatively affect people’s willingness to speak about their concerns in the workplace (Sarfraz *et al.*, 2021; Morrow *et al.*, 2016). Status differences and asymmetrical distributions of resources (such as rewards, reputation or recognition) can make it difficult or too risky for employees to speak up about problems (Kritsotakis *et al.*, 2022; Morrison, 2023). Furthermore, voice and silence can themselves serve as a means of exerting or resisting the use of power (Morrison, 2023).

As noted above, a culture of “open communication” at work has been explored from various angles, primarily focusing on how things are (factual based) or how they should be (normative), rather than how things are “talked into being” (discursive approaches). This study expands on previous research by regarding “open communication” as a contemporary discourse of work, which, we argue, represents the prevailing ideology within the social structures of our societies. By the term “ideology”, we refer to a system of dominant ideas and beliefs that affect every sphere of human social interaction and organization (Zajda, 2014). It is assumed that when people “explain, motivate or legitimate their (group-based) actions, they typically do so in terms of ideological discourse” (van Dijk, 2006, p. 121). 

In the following, we employ discourse analysis to examine nursing professionals’ team discussions about problematic interaction situations in their work, which invoke and draw upon the discourse of the “open communication culture”. We ask: (1) What properties are consistently used to describe “good” and desirable interactional behavior among colleagues in problematic situations, and (2) how do nursing professionals reproduce the dominant interpretations of “good communication” even in situations that highlight the practical problems of realizing these ideals? Through our empirical analysis, we demonstrate how nursing professionals retrospectively account for problematic interactional situations and how they position themselves and their colleagues within these accounts during team discussions, with both their colleagues and a nursing supervisor present.

## Materials and methods

As data, we used workshop discussions from 14 teams who participated in a work development process related to ethical stress and work culture. All teams were located in four Wellbeing services counties in Finland. The workshops were organized as part of the “*anonymized*” project, which is an implementation project of the *anonymized*, funded by the *anonymized*.

The four participating counties were municipal authorities responsible for arranging social and healthcare services in their respective areas. The participating units represented three different care concepts for the elderly population. *Home care* units provided healthcare and supportive care services in the clients’ individual homes. The *service housing* units were meant for clients who needed care services around the clock, for example, clients with dementia or a progressive physical illness. The *hospital nursing wards* mainly had palliative or hospice care patients.

The workshop process lasted three to five weeks in 2021 and involved a combination of in-person and video conferencing workshops, depending on the regional COVID-19 restrictions at that time. The workshop process in every unit consisted of two workshop meetings. In the first meeting, the current state of employee well-being was discussed, and the goal was to identify ethically problematic situations at work. In the second workshop, the aim was to seek solutions to these problems and design experiments to be carried out at workplaces. The workshop discussions were audio recorded and transcribed verbatim.

Each workshop had one team with 7–20 participants: one unit supervisor (*n* = 14) and 6–19 care workers (*n* = 142), totaling 156 participants. The unit supervisors all had nursing training and over 15 years of work experience in elderly care. They all participated in the operative clinical work. Most of the care workers were practical nurses who worked to a three-shift schedule. We had no information on their age, gender, or work experience. As the workshops aimed to improve organizational work practices, the participants were recruited from within the organizations, with no research-based inclusion or exclusion criteria. As each workshop involved participants from the same team, the participants were familiar with each other. The facilitators were well-experienced researchers and teachers in work life development and nursing education.

Permission to collect the data was obtained from the organizations and the Ethics Committee of *anonymized* (decision *anonymized*). Informed consent was obtained from all the participants, and they were advised of their right to withdraw their consent at any point during the data collection. To ensure confidentiality and privacy, and to avoid any group pressures, consent was given privately to the researchers, who only recorded the workshop if all participants gave their consent. If even one participant did not give consent, the workshop proceeded without data collection. Considering the sensitive nature of the discussions, the facilitators emphasized the confidentiality of all discussions within the workshops among all participating team members and throughout the research process. In the analysis, we removed or altered all the identifying details of the participants in the text.

Methodologically, our data analysis drew from discourse analysis (e.g. Potter and Wetherell, 1987). Discourse analysis is based on following theoretical assumptions (Jokinen *et al.*, 1993): (1) Social reality is constructed in and through language and the ways in which its use results in tangible consequences. (2) Social reality is both born and continually transformed through language, which enables the transformation of social reality and individuals to align themselves with reference to these constructed social realities in different ways. (3) Meanings are inherently context specific and arise as individuals engage with the objects and entities in the situation. In our research, this meant that we focused on how the nursing professionals who discussed the development of ethical workplace culture used language to construct their ideals about how a competent nurse should behave in interactionally problematic situations. By “interactionally problematic situation” we refer to interactional encounters with unsatisfactory or unsettling outcomes that have the power to bother the participant afterward (Cui, 2014).

In the data-driven analytic process, three researchers (*anonymized*) first identified segments of talk in which the nursing professionals talked about interactionally problematic situations. Next, the first (*anonymized*) and the second (*anonymized*) authors separately conducted qualitative case-by-case analysis, focusing on the regularities in language use. As ideological discourses are typically constructed in and through explanations, motivations, and legitimizations (van Dijk, 2006), we were interested in how the nursing professionals retrospectively thought the situation should have been handled. These retrospective accounts could be characterized as social actions by which people “make sense of their words and (perhaps) impose that sense on other people” (Antaki, 1994, p. 1). In addition, we were interested in how the nursing professionals positioned themselves and their colleagues in their accounts. Positioning can be considered “dynamic and evolving clusters of norms and expectations” that people perform (or reject) in particular moments of interaction in varied and unique ways (Green *et al.*, 2020, p. 121). The case-by-case analyses were jointly discussed with the whole research team to research the consensus. These discussions led to dividing the cases into categories (four imperatives for being a good team member) that we had jointly agreed upon and that we could reliably identify in the data. Lastly, the first (*anonymized*) and the second (*anonymized*) authors analyzed the nursing professionals’ resistance and reproduction of the discourse and its ideals. In this analysis, we exploited the concept of *counter-talk,* which refers to the implicit or explicit disruptions of the conventional flow of talk, such as deviations from the topic, disagreements, or counter-questions, and allow the researcher to examine the power relations and moral boundaries presented by the participants of the interaction (Heikkilä and Katainen, 2021). This analysis was again discussed together leading to several further speciﬁcations in the final analysis.

In the analysis section below, we describe the patterns of nursing professionals’ language use that we identified across our whole data set, with the help of data extracts that we chose to illustrate the phenomenon in a clear and accessible way. The Finnish transcriptions were translated into English by the authors.

## Results

When the nursing professionals discussed interactionally problematic situations in their workplace, they described their interactional conduct with reference to the ideal of being a “good”, competent team member. Such accounts invoked and drew from the discourse of “open communication”, which is constructed and oriented toward as normatively binding. In the first subsection below, we delineate the properties of this discourse, showing how the nursing professionals predominantly reproduced its ideals. In the second subsection, we discuss cases in which the ideals of “open communication” were acknowledged as leading to problems, but in which the apparent challenges to the dominant discourse were nonetheless silenced.

### Being a competent team member

The nursing professionals described the preferred interactional conduct among their colleagues in problematic situations using four imperatives: (1) Engage in direct communication and avoid making assumptions, (2) Address problems immediately, as soon as they emerge, (3) Refrain from gossip, and instead deal directly with the person involved in the matter, and (4) Summon the courage to speak up. Below, we explain the orientations to each of these normative imperatives using data examples and highlighting their ideological character.

#### Engage in direct communication and avoid making assumptions

When discussing challenging workplace interactions, the nursing professionals adopted a normative position according to which it was unacceptable to make assumptions about their colleagues’ thoughts or feelings during these encounters. The quotation below demonstrates the professionals’ orientations to this normative ideal. Here, one of the participants has shared a recent real-life experience about colleagues who had exchanged angry looks instead of addressing their problem directly.

Don’t engage in any staring contests. If someone believes they’re staring at you and assessing you, it’s not a good idea to stare back; instead, just ask them openly “excuse me, is there something we need to talk about?” It may just be their way that they stare a bit but then you draw your own conclusions, rather than discussing things right away. Then later it keeps bothering you.

The nurse position themselves as a competent team member who understands the appropriate interactional conduct and can offer guidance. The nurse advises against engaging in a “staring contest”, when one professional glares at another. Instead, the professional being stared at should openly ask if there is a problem that needs discussion. Engaging in open discussion enables them to avoid forming hasty judgments about their colleague’s look and prevents any subsequent lingering thoughts. Here, “staring a bit” is considered a personal habit and is not inherently problematic. What renders the interaction situation problematic is the other person’s inclination to “draw your own conclusions”. The problematic nature of the inclination to draw conclusions is explained by the initial problem later “bothering you.”

The participants also positioned themselves as not being able to interpret their colleagues’ indirect communication cues. In the quotation, the speaker, a ward manager, expresses frustration with nurses in the ward who expect the manager to be able to decipher the “true meaning” behind their words.

In a way, I have to deal with that assumption-making myself. I always think that the employees sort of expect me to read between the lines, that someone says something very subtly, and I might not necessarily catch it. It doesn’t mean I’m not paying attention; I simply didn’t realize that the issue as it was mentioned in an aside. But some employees might assume that they’ve already informed me of it, creating a contradiction where they believe they’ve communicated something to me, but I just haven’t taken it in. And in this situation, it’s not intentional ignoring; I just didn’t get it.

The manager holds their subordinate nurses accountable for their indirect communication, which falls short of the ideal of direct communication. By conveying their message “between the lines” and briefly mentioning crucial matters “in an aside”, the nurses are relying on the manager being able to read subtle interactional cues and understanding what they have intended to convey. By casting the interactional problem as a mere problem of understanding, the manager avoids any responsibility for problematic events (Stevanovic, 2023). It is the nurses who should have behaved differently, enabling the manager to “get” their point.

In sum, the professionals focused on the need to concentrate on the explicit content of what was being said in the interaction, to pose direct questions when something was not clear, and to avoid making assumptions about others’ hidden intentions behind their conduct. Such assumptions were presented as even more problematic than the initial interaction problems.

#### Address issues immediately, as soon as they emerge

The nursing professionals oriented to the norm that, when an interactional issue arises, a competent participant should be able to intervene immediately. The third data example is from the same conversation as the first one. Here, the nurse once again refers to the staring contest at work and admonishes their colleague for not having addressed the problem immediately.

I asked this [colleague], “Why couldn’t you have just politely asked, excuse me, but do you have something you want to talk about as you’re staring so intensely?” You could have gone through it right away and talked about it instead of engaging in a staring contest. Because they could have addressed the issue immediately, instead of leaving it as a staring contest where one thinks they’re being judged. Just openly ask “Sorry, but do you have something on your mind, or is there something we should talk about?” So, that’s what I did in this case. We talked about the importance of addressing things directly, right away.

The nurse’s description portrays immediate intervention as the ideal course of action and recounts how they questioned their colleague’s conduct during the situation by inquiring why the problem was not addressed on the spot. In this way, the nurse who complained about their colleague’s staring in retrospect was held accountable for not addressing the issue immediately. The speaker also positions themselves as a competent team member who did address their colleague’s complaint as it arose by directly challenging the colleague’s lack of prior intervention.

The nursing professionals constructed immediate intervention in interactionally problematic situations as an effective way to guide and educate colleagues in appropriate behavior.

S1: It should be addressed immediately, when it happens. You wouldn’t scold a dog a week after it peed on the floor. So, it’s just right away, it should be for everyone …

S2: That’s when it has the most impact.

The first speaker, S1, takes a normative stance, emphasizing that everyone should intervene immediately when problems occur. S1 uses a culturally recognizable example to illustrate ineffective ways of modifying someone’s behavior: likening it to scolding a dog a week after it has peed on the floor. S1 underscores the significance of immediate action and claims that this principle should be clear to everyone. S2 agrees with S1, asserting that immediate action has “the most impact”.

To conclude, the nurses constructed immediate intervention as a normative ideal. If team members failed to intervene immediately and addressed the problem retrospectively, they were expected to admit their failure. Additionally, immediate interventions were considered useful for educating colleagues on how to “behave correctly”. In contrast, the question of whether everyone should have the authority to educate their colleagues was avoided.

#### Refrain from gossip and deal directly with the person involved in the matter

It was deemed essential that the problems are discussed with the person directly involved in the matter. The next extract exemplifies this. Here, the speaker summarizes the small-group discussion to all the workshop participants.

We also talked about the importance of giving feedback openly and discussing issues. And discussing them with the person concerned. So, for example, if a relative or a client tells one employee something negative about another employee, the story tends to continue and may change along the way.

The speaker addresses the importance of discussing negative feedback directly with the person concerned. The speaker highlights the potential risk of the feedback’s content changing or the problem persisting if it circulates among the employees. In addition, voicing complaints about colleagues was presented as personally threatening, as demonstrated below.

So, if someone criticizes or says something negative, it makes me think “Why don’t you say it directly to that person or something?” But you don’t dare say at the coffee table that you don’t want to listen to that person yapping, and “Why don't you go and talk to that person instead?” Because they might criticize me, and then I wouldn’t belong to this great group.

The speaker recounts an incident where a colleague, as described by the speaker, criticizes another colleague behind their back in a coffee break room. The speaker regards this behavior as “yapping” and suggests that a person who is badmouthing their colleague should address the problems directly with that person. The speaker also envisions speaking up to this colleague and revealing their unwillingness to tolerate the “yapping”, but does not dare to do so because of a fear of potential exclusion from the group.

In sum, it was deemed essential that any problems were dealt directly with the person involved in the matter. Discussing problems with anyone else was seen as dragging out the problems. Moreover, discussing problematic events with someone else invoked the culturally negative idea of “gossiping”.

#### Summon the courage to speak up

“Open communication” was framed as both an interactional skill and a personality trait that requires courage. Those who did not “speak up” were blamed for problematic situations, with the prevailing belief that encouraging everyone to voice their concerns would help resolve interactional issues. The next conversation example illustrates this.

S3: I think that if there are issues …

S4: Then speak up.

S3: That’s right.

S4: Ask for help, talk, discuss.

M1: Sure, if there’s a problem, I feel like you’re all capable of solving it. You can certainly tell a colleague “you can’t do that, this is what we do or you should think about how you’re going to do this” … Of course, in a female dominated field there's always some talking behind each other’s backs, and that probably never stops, because we’re women and maybe men are better at giving feedback directly. Sometimes we might beat about the bush, and there probably could be more of that direct feedback.

The nurses (S3 and S4) show a consensus that addressing issues requires speaking up if problems are to be resolved. The department manager (M1) joins the discussion, asserting that the nurses are fully capable of managing problematic issues among themselves. The manager provides examples of how a nurse can, for instance, instruct a colleague not to do something. Thus, the manager positions them as educators responsible for guiding their colleagues. Next, the manager suggests that the nurses could give more direct feedback. They link the predominance of females in the nursing field to the perceived “indirectness” in communication, suggesting that males, in comparison to females, are better at delivering direct feedback.

The absence of directness was also attributed to a lack of courage. Furthermore, this lack of courage was presented as something to be remedied by acquiring the required skill. This orientation is demonstrated in the following quotation where the ward manager has mentioned that their unit has individuals who have expressed that their way of doing things is the only right way. In the quotation, the manager explains that different styles of doing things does not require the intervention of a supervisor.

But also, the fact that we didn’t really have courage to openly speak up or talk about things, and now there’s been a strong effort to learn that. I’ve been trying to tell everyone that you don’t always need me to resolve every issue, that you could consider discussing things among yourselves.

The ward manager emphasizes the significance of nurses independently addressing and resolving problematic situations. Here, the absence of “directness” is attributed to a lack of the courage required to voice concerns about problematic issues. Additionally, nurses are portrayed as individuals who must develop greater courage to enhance their ability to speak up properly.

In conclusion, the courage to speak up was framed as a moral duty for individual nurses, allowing them to become more effective team members and enabling an immediate resolution of problematic issues among themselves. The nurses were deemed accountable not only for their proficiency in teamwork skills, but also for their responsibility to educate others. The courage to speak up was furthermore associated with gender stereotypes, and a lack of “directness” attributed to females. This stereotype was used to rationalize the failure to achieve the ideals of “open communication”.

### Commitment and counter-talk for an open communication culture

Even when the nursing professionals questioned their abilities to commit themselves to the imperatives of “open communication” in everyday workplace interactions, these imperatives were consistently presented as normative ideals toward which to strive. The data extract shows an example of this. Here, a small group of people are summarizing their thoughts on how to make their workplace culture more ethical.

S5: Could it be noted that we refuse to gossip, and talk about others behind their backs? Let’s discuss the matter with the person involved first, or something? It’s really difficult to start, in a group, you sit, you listen, and when something is going on, it’s difficult to say something, because otherwise,

S2: I understand completely, it’s not easy.

S1: Then someone stops belonging to that group, that’s how it goes.

S2: This should be made the culture of the whole work community.

S5: Yeah, that’s kind of what I mean. So that everyone, even if they don’t say anything, would, maybe, walk out or I don’t know. It’s easy to say, of course.

S6: There will never be such a work community.

S5: Probably not, no. Should I include it at all then?

S2: Just include it! Because it’s concrete, a very important thing.

Speaker 5 proposes writing down one ideal of “open communication” discussed above, namely resolving problems directly with the person involved. However, after bringing up this ideal, they reflect on the difficulty of realizing it in real life: A group situation makes it difficult to speak up. Two other participants agree with S5, stating that speaking up risks becoming excluded from the group. To address this problem, another speaker, S2, suggests that “speaking up” should become the “culture of the whole workplace”. S5 agrees and admits that, while “it is easy to say”, the ideal may be difficult to realize in practice. Also, S6 acknowledges this difficulty, stating that there is no such workplace where speaking up is easy. S5 agrees and proposes the group to exclude this ideal in their summary. However, the proposal is rejected, as the issue of “speaking up” is considered a “very important thing” to include. Thus, the participants commit themselves to the ideal of an “open communication culture”, even if they find it challenging to implement in real-life.

In rare cases in our data, some of the nursing professionals positioned themselves as resisting certain aspects of the “open communication culture”. In these instances, they expressed counter-talk against the ideal of speaking up, emphasizing that it was the colleague’s misbehavior that was morally questionable, and not their lack of ability to address the questionable behavior. This is the case in the last example. In the first lines of the example, speakers 2 and 5 promote the “open communication” ideal, after which S1 challenges it.

S2: The work community should be one in which you dare speak about things.

S5: Like openness.

S1: “Cause I think, what then, if you do say something, or somehow, then it doesn”t really change anything anyway. If it leads to a change once, I don’t think the employee really has any capacity for any more.

S3: It’s frustrating because you end up having to deal with the same issue every time.

S1: Yes, that’s true, yeah.

S2: If there’s no change.

S1: Because an employee can’t really get into someone else’s morals or ethics. S4: If everyone always remembered that you can argue about issues, but the argument doesn’t have to be personal. We can have different opinions on things and so on, without having to start fighting about it.

S1 initiates counter-talk by claiming that “speaking up” in problematic situations fails to make any difference. Even if a colleague changes their behavior temporarily, one person alone cannot bring about a lasting change in the colleague’s behavior. S3 acknowledges this frustration that arises when an employee repeatedly must deal with the same problematic behavior. S1 then suggests that the issue at hand concerns a poorly behaving colleague’s moral and ethical values. The poor behavior is morally questionable, not the other person’s (in)ability to address it. Next, S4 uses the idiomatic expression “issues conflict, not people”, which serves to close the discussion (Drew and Holt, 1998). In opposing the need to avoid conflict to safeguard social relations, S4 reconstructs the ideal that conflictual views should be openly expressed. This effectively silences S1’s counter-talk.

## Discussion

In this article, we have shown nursing professionals’ discussions on problematic interactional situations in their work, and how they consistently invoke and draw upon the discourse of the “open communication culture” ideal. In this way, our study contributes to the existent literature on how, when, and to whom one should speak up at work (Kane *et al.*, 2023; Okuyama *et al.*, 2014). We have described nursing professionals’ orientation to the discourse of “open communication”, using four imperatives as a reference. Following these imperatives helped us define “good” and desirable interactional behavior among colleagues in problematic situations. Even when commitment to the imperatives of “open communication” was considered difficult to realize in real-life workplace interactions, these imperatives were presented as normative ideals toward which to strive and any counter-talk resisting the dominant discourse was silenced. The need to comply with these ideals was nonetheless constructed as self-evident, which highlights the ideological nature of the “open communicative culture”.

As noted in the introduction, “speaking up” is connected to employees’ diverging experiences of psychological safety in the workplace (Frazier *et al.*, 2017; Grailey *et al.*, 2021). This might have been the case, for example, in Extract 11, in which one professional’s experiences of speaking up differed from those of the others. In this case, the professional did express their diverging view but let it pass when it was disregarded by the others. In some other cases in our data, we did not know if some of the professionals who resisted the construction of the shared view of “open communication” felt insecure in voicing their disagreement. This type of “speaking up” on a meta level (speaking about the collective norms and ideals of speaking up) also requires courage, and thus may be easier for some people than for others. We further suggest that “speaking up”—and particularly the norm of always speaking up—may not in itself advance psychological safety at work. Instead, acknowledging the interpersonal risks involved in “speaking up” (e.g. Sarfraz *et al*., 2021; Pearsall and Ellis, 2011) and increasing awareness about the threats to “face” associated with direct communication (see Goffman, 1955) can promote genuine experiences of psychological safety.

Speaking up and voicing one’s concerns directly to those who are involved in the matter is a widely acknowledged norm in working life (e.g. Morrow *et al.*, 2016). What might be specific to the nursing profession in this respect is the centrality of interaction in their work. It is widely acknowledged that nursing professionals should possess excellent interaction skills (e.g. Gur and Tzafrir, 2022; Anderson *et al.*, 2014). Consequently, problems related to workplace interaction may be relatively difficult to acknowledge. When colleagues point out deficiencies in each other’s interaction skills, they may be easily interpreted as questioning each other’s professional competence.

Furthermore, it can be argued that in the nursing profession, the norm of open communication serves to uphold the core value of the profession, which is the wellbeing of patients (Kane *et al.*, 2023; Okuyama *et al.*, 2014; Bliss *et al.*, 2017). In our data, the nursing professionals framed “speaking up” as something straightforward with no significant risks of interpersonal conflict. However, this perspective seems somewhat oversimplified, especially when the issues to be addressed are of a moral nature. This is frequently the case with nursing. Previous research has suggested that nurses’ perceptions of “a good nurse” align with virtue ethics, in which a person’s actions are believed to reflect their character (Numminen *et al.*, 2017; Aydin *et al.*, 2017). If “doing what is right” and “being good” are intertwined, the line between criticizing a colleague’s actions and questioning a colleague’s ethics can become blurred. Challenging the morality and “goodness” of a colleague is difficult to present as neutral criticism. This may partly explain the incongruity between professionals’ ideals and actions in our data and reinforce the notion that direct “speaking up” may not always be advisable in interactionally problematic situations. Instead of voicing problematic interactional events *directly*, *immediately,* and *to those concerned*, professionals may choose to let problems pass, normalize them, and talk about them behind people’s backs (see Visuri *et al.*, 2023; Koskela *et al.*, n.d.). Although they depart from the ideal of speaking up, the above-mentioned risks justify these responses.

In our data, it was the ward managers in particular who framed the resolutions of interactional problems as a matter of individual nurses improving their interaction skills and guiding their colleagues toward morally desired professional conduct (see Extracts 2 and 8). At the same time, the managers themselves avoided being held responsible for the problematic events (Stevanovic, 2023) and mitigated their role in the management and retrospective addressing of the interactionally difficult situations. In this way, our findings also highlight the need to recognize leadership styles and the existence of interactional inequalities. Not all interaction participants are held accountable for their interactional transgressions in the same way, and to emphasize the need to address such transgressions immediately only reinforces these inequalities: it is the powerful who actually have the resources to fulfill the norm, while the powerless carry the double burden of only daring to grumble about the transgressions in retrospect and feeling guilty about this retrospective grumbling (Stevanovic, 2023). As it is the participants in lower hierarchical positions who typically remain silent for valid social reasons (e.g. Morrison, 2023; Kritsotakis *et al*., 2022; Lam and Xu, 2019), managers appealing to the prevailing ideologies of “open communication” in effect only strengthen their position of power in relation to their subordinates. Consequently, collective leadership with low hierarchical relationships and a focus on continuous learning of collective capabilities in patient care may be beneficial for enhancing a genuinely open communication culture.

Further research is needed to explore the dynamics of workplace communication, particularly focusing on how power imbalances affect individuals' ability to express themselves. This could involve studies on micro-level communication patterns related to power negotiations, interventions to empower marginalized voices, or the development of communication strategies that promote inclusivity.

### Limitations

Our research was limited by the lack of demographic characteristics of the participants. We lacked details about each participant’s specific role in their workplace, and only those who explicitly assumed managerial positions during the discussion could be reliably identified as such. This limitation reduces our possibilities to assess the representativeness of the sample. As the participants were recruited from within the organizations without research-based inclusion or exclusion criteria, recruitment may have introduced a bias, as individuals who volunteered to participate might, on some dimension, differ systematically from those who did not. As each workshop was recorded only if all team members gave their consent, it is possible that individuals in teams with especially challenging relationships were less likely to consent. Consequently, it is possible that our results about the nursing professionals’ orientations apply most accurately to relatively well-functioning teams.

Due to COVID-19 restrictions at the time of the data collection, some workshops were conducted via video conferencing. This may have affected how participants interacted, as their limited access to each other’s non-verbal behaviors is known to pose challenges in producing, interpreting, and coordinating dialogue, especially on affective topics (e.g. Büyükgüzel and Balaman, 2023). But then again, the participants were familiar with each other and accustomed to working via video conferencing. The workshop processes were also relatively short. Two workshops may not have provided sufficient time for the participants to delve deeply into complex topics. While this study did not investigate the efficiency of the intervention, we may assume that such a short duration might not have allowed for a comprehensive exploration of the team’s work culture.

Collecting data from team discussions required the nursing staff to describe their experiences in the presence of their supervisor. This power dynamic may have influenced how the nursing staff addressed difficult topics at work, particularly those related to the supervisor. Organizing separate workshops for the nursing staff and the managerial staff may have been beneficial for collecting information about each group’s perceptions of problematic workplace interactions. Additionally, enriching the workshop data with interviews or other data sources could have provided multiple perspectives on the same phenomenon, thereby increasing the validity of the results. However, our current data set had other advantages. The recordings of discussions among authentic team members provided us with exceptional access to how team members not only discussed problematic workplace interactions and the ideals of open communication but also how they enacted them *in situ*. Hence, it allowed a more nuanced consideration of power relations and the ways in which dominant ideologies were reinforced in the moment-to-moment social interactions among teams. Given that social interactions between managers and employees have been shown to significantly influence the formalization of expectations regarding normatively “good” interactional behaviors (Frazier *et al.*, 2017), our study could show in detail how this happens. Future studies, however, should systematically explore the potential differences in how employees and managers invoke and draw upon the discourses and ideologies of workplace interaction.

### Implications for practice

The significance of this study for practical workplace development lies in how it illustrates the way in which the workplace environment, coupled with our comprehension of societal discourses, molds our perceptions of the ideals of workplace interaction. These ideals, in turn, influence individuals’ behaviors in diverse situations. More specifically, our findings highlight the necessity of understanding how the norm of “open communication” operates in the nursing profession, unveiling the distinctive characteristics of the healthcare sector in this regard. Although the normative emphasis on “speaking up” may prove beneficial for enhancing patient safety (e.g. Kane *et al.*, 2023), it may not be as well-suited for fostering collaboration and the emergence of a psychologically safe work environment among colleagues. Compared to the voicing of ideas, suggestions, and observations about work-related issues as an attempt to bring about improvement, calling into question the productivity of colleagues’ work behavior poses a risk for employees and their social relations in the community (Morrison, 2023). Consequently, the expectation that an individual will simply “speak up” when they experience mistreatment by a colleague might be too much if that individual is already in a vulnerable and precarious position. Hence, we propose that workplace communication should be enhanced at a communal level, allowing those with less power to express their perspectives on shaping shared ideals of workplace interaction—or to choose not to speak up. This development might involve implementing policies and practices that foster open communication, providing training on active listening and respectful dialogue, or restructuring hierarchies to distribute power more evenly.

Enhancing workplace communication at a communal level can even have broader societal implications. By empowering individuals with less power to express their perspectives, organizations contribute to greater inclusivity and diversity in the workplace. This can lead to increased employee satisfaction, productivity, and innovation (e.g. Hämmig and Vetsch, 2021), ultimately contributing to a more equitable society.
